# The Pandemic of Hate is Giving COVID-19 a Helping Hand

**DOI:** 10.4269/ajtmh.20-0285

**Published:** 2020-04-20

**Authors:** Edmond Ng

**Affiliations:** London School of Hygiene & Tropical Medicine, London, United Kingdom

Chinese restaurants are losing business in America. France faces an “epidemic” of anti-Asian racism. #ChineseDon’tComeToJapan is trending on Twitter.

What began as a small cluster of pneumonia of unknown cause in Wuhan, China, in late 2019 has now become one of the deadliest pandemics the world has seen since the 1918 flu pandemic. The novel coronavirus, severe acute respiratory syndrome coronavirus 2 (SARS-CoV-2), has brought the most severe challenge to the global economy and to stability since the World War II. In addition, it has unleashed a raging pandemic of hate and widespread stigmatization, especially against the Chinese and East Asian diaspora, in many countries.

Sadly, London, the city where I live and work, is no different. From the physical to the verbal, abuse toward these communities, including some of my friends, our staff, and students at the London School of Hygiene & Tropical Medicine, is seen. For example, in my 9-year-old daughter’s primary school, children were spreading rumors that a mixed-race child, who is of Chinese origin, was spreading the coronavirus. A stranger shouted “you are a virus” at the husband of a colleague visiting from mainland China, before shoving their family, including their two young children, down the stairs at a London Underground station. She was traumatized by further encounters of racial abuse and decided to give up her research in London to return home with her family. Frontline doctors of Chinese origin have been verbally abused, including one being told by a patient that the virus came from his kind—Chinese.^1^

Racist and violent acts have not been limited to London; reports of similar events have been filed from around the world, with just a few examples cited here.^2–4^ In the United States, more than 1,000 media reports of racism and xenophobia against Asian Americans were recorded between January 28, 2020 and February 24, 2020, as reports of cases of coronavirus infection began to emerge in the United States.^5^ The rapid increase in the frequency of such incidents led the National Council of Asian Pacific Americans to send a call to the Congress to denounce anti-Asian racism around coronavirus.^6^

Novel coronavirus disease (COVID-19)–driven xenophobia has now begun to spread beyond anti-Asian sentiments; in Guangzhou, China, fears of importation of COVID-19 prompted landlords and hotel managers to evict Africans, despite many claiming to have no recent travel history or known contact with COVID-19 patients.^7^ This is reminiscent of the anti-African sentiment during the 2013–2016 West African outbreak of Ebola virus disease.

The racism and xenophobia are not limited to the streets; incendiary rhetoric like “the Chinese virus” and “China’s fault” have shamefully been adopted by some prominent public figures and fora, providing the necessary oxygen to fan the flames of hate and discrimination against members of the targeted communities.^8^ It serves as a distraction that helps politicians evade scrutiny of pressing local issues. The headline “Alerte Jaune” (“Yellow Alert”) carried by a French newspaper is reminiscent of concerted misinformation campaigns during the European conquest of the “backward” exotic Far East.^9^ This rhetoric also helps embolden the far right to pursue their anti-immigration agenda and jeopardizes the world’s progress in tackling stigma, while undermining international collective efforts to slow the pace of the accelerating pandemic.

From the spreading of syphilis in Europe and Asia in the 1500s, during which each affected country blamed their neighboring countries or enemies for their outbreak,^10^ to the scapegoating of the Chinese community as a source of the foot-and-mouth outbreak in the United Kingdom in 2001,^11^ xenophobia and racial prejudice have been associated with infectious disease outbreaks. Such behaviors are often driven by ignorance and fear of the nature and spread of the disease, and they are fueled by rumors and misinformation.

Some reactions may have roots in lack of awareness and understanding of historical events and cultural norms. The use of face masks is a prime example; the sight of East Asians wearing face masks on public transport and in communal place has caused panic and become a catalyst for rising levels of fear in some Western cities.^12–15^ Some of the attacks on Chinese students in the United Kingdom were reported to have been triggered by the so-called maskaphobia.^12^ The deadly toll of SARS and MERS left an indelible collective traumatic memory in East Asia.^15^ People outside of East Asia may not realize that in these countries face mask-wearing is perceived as a civic responsibility to avoid disease spreading, and even a symbol of defiance in their fight against deadly diseases.

Regardless of our race or ethnic background, and whether targeted individually or not, we all suffer from these acts of hatred. In mid-March 2020, Chinese students were reported to be fleeing the United Kingdom en masse, partly as a result of increasing levels of racially motivated hostility and attacks. Some expressed their fear of racism being worse than their fear of the virus itself.^12^ It is reasonable to expect such reactions from those who are targets and victims of racism.

As we flee to our separate global corners, we lose cultural awareness and the opportunity for a rich exchange of ideas and knowledge on art, language, science, and economics. Importantly, we also impede the very biomedical advances needed to fight threats such as COVID-19. Students and researchers who experience such open hostility overseas may think twice about returning to some of the top academic institutions to pursue scientific discovery, resulting in an acute loss of diversity in the higher education and research communities in which high-quality international research thrives. The economic impacts may also be profound; according to one estimate, British universities are at risk of a funding gap upward of 200 million pounds because of hesitance to enroll for the new academic year among Chinese students.^16^

Novel coronavirus disease is claiming victims because of not only its transmissibility and virulence but also its ability to spread fear and ignorance. We must deny stigma and racism a foothold to foment hate and division, and replace it with unity across all communities to spread empathy and compassion, and to strengthen our collective resolve to defeat COVID-19 together. Viruses do not discriminate based on race, religious belief, gender, or sexual orientation, and nor should we. We should be fighting the virus, not each other. As António Guterres, secretary-general of the United Nations, said, “Now is the time for unity.”^17^
